# Diagnosis of congenital Hyperinsulinism can occur not only in infancy but also in later age: a new flow chart from a single center experience

**DOI:** 10.1186/s13052-020-00894-5

**Published:** 2020-09-14

**Authors:** Alberto Casertano, Arianna De Matteis, Enza Mozzillo, Francesco Maria Rosanio, Pietro Buono, Valentina Fattorusso, Adriana Franzese

**Affiliations:** Department of Translational Medical Science, Section of Pediatrics, Federico II University of Naples, Via Sergio Pansini 5, 80131 Naples, Italy

**Keywords:** Congenital Hyperinsulinism, ABCC8, Hypoglycemia, Diagnostic flow-chart

## Abstract

**Background:**

Congenital Hyperinsulinism typically occurs with a neonatal hypoglycemia but can appear even in childhood or in adolescence with different types of glucose metabolism derangements. Current diagnostic algorithms don’t take into account cases with a late presentation.

**Patients and methods:**

Clinical and laboratory data of twenty-two subjects diagnosed at Federico II University of Naples have been described: patients have been divided according to the molecular defect into channel defects, metabolic defects and unidentified molecular defects. A particular focus has been made on three cases with a late presentation.

**Results and conclusions:**

Late presentation cases may not be identified by previous diagnostic algorithms. Consequently, it seems appropriate to design a new flow-chart starting from the age of presentation, also considering that late presentation cases can show glucose metabolism derangements other than hypoglycaemic crises such as diabetes, glucose intolerance, postprandial hypoglycaemia and gestational diabetes.

## Background

Congenital Hyperinsulinism (CH), first defined by Stanley [1], represents the most common cause of persistent hypoglycemia (HY) in infancy with estimated incidence of 1:40.000–50.000 in general population, up to 1:2500 in case of consanguinity [2]. To date, its prevalence in Italy is not well known [3, 4]. CH is due to alteration of beta-cell membrane channels or intracellular metabolic pathways, involving insulin secretion. In particular, channel defects (ChD), deriving from mutations of the K-ATP channel, represent about 36% of all CH cases [5] and affect *ABCC8* and *KCJN11* genes, which codify respectively for Sulfonylurea Receptor 1 (SUR1) and Kir6.2 (that are K-ATP channel subunits). Metabolic defects (MeD) are due to defective beta-cell intracellular signaling. To date, 12 genes are known to be responsible for MeD cases [5, 6]; GCK and GLUD1 are the less rarely involved genes. CH is suspected in case of neonatal onset of persistent HY with inappropriately measurable blood insulin levels. A further diagnostic criterion can be an intravenous glucose requirement of more than 8 mg/kg/min [7, 8]. The flow-chart of Maiorana et others [9] proposes to start with metabolic screenings and states that ^18^F-DOPA PET should be performed in all cases.

It is known that the diagnosis of CH can also occur in adolescents and adults reported for symptoms other than HY, such as mild hyperglycaemia and gestational diabetes mellitus (GDM) [10–14], thus justifying a late diagnosis of CH [15].

In light of our clinical experience, we propose a new flow chart, which represents an extension of the diagnostic range already presented by others in the literature with the advantage of making the diagnosis quicker and more accurate.

## Patients and methods

Twenty-two patients, who received the diagnosis of CH and were followed for at least 3 years at the Regional Center for Pediatric Diabetes of the Federico II University of Naples, are reported in our study.

Diagnosis of CH was based on the detection of measurable insulin levels during the hypoglycaemic crises starting in neonatal age/infancy. The molecular diagnosis was performed according to the current diagnostic flow-charts in neonatal cases; an accurate clinical investigation was essential for the diagnosis of the three late presentation cases.

Based on their genetic defects, patients have been classified into three groups: ChD, MeD and unidentified molecular defects.

The data collected are: HY and other glucose metabolism derangements (GMD) in the family, large weight at birth (LGA) and delivery at term or preterm, HY onset before or after 72 h from birth, responsiveness to Diazoxide (Dx), surgical treatment and, in the follow up, presence of diabetes, obesity and neurological impairment. Data from ChD subjects (*ABCC8*-mutation) has been reported in Table 1, data of MeD and unidentified defects in Table 2.

**Table 1:** *ABCC8* mutated patients (ChD)

	Sex	Genotype	Family History		Birth		HY Onset		Treatment		Follow-Up			
			Hypoglycaemia	Diabetes (IFG/IGT)	At Term	LGA	< 72 h	Postneonatal	Dx responsiveness	Surgery	DM	Obesity	Neurological Impairment	Duration (years)
**1**	**M**	**Maternal Dominant** **c.G4435 > A (p.G1479R)**	+	+	+	+	+	–	–	–	+	–	–	6
**2**	**M**	**Maternal Dominant** **c.G4435 > A; (p.G1479R)**	+	+	–	+	+	–	–	–	+	–	–	3
**3**	**M**	**Maternal Dominant** **c.4616G > A; (p.R1539Q)**	+	+	+	+	+	–	+	–	–	+	–	6
**4**	**M**	**Maternal Dominant** **c.3088G > T; (p.D1030Y)**	–	+	+	+	+	–	+	–	–	+	–	8
**5**	**F**	**Paternal Dominant** **c.172G > A; (p.V58M)**	–	–	+	–	–	+	+	–	–	+	+	22
**6**	**M**	**De novo Dominant** **c.4519G > A; (p.E1507K)**	–	–	+	+	+	–	+	–	–	+	–	22
**7**	**M**	**De novo Dominant** **c.3133_3152del2; (p.T1045fs)**	–	–	+	–	+	–	–	+	+	–	+	20
**8**	**M**	**Dominant** **c.1333-1013A > G (**^**a**^**)**	–	+	–	–	+	–	+	–	–	–	–	6
**9**	**M**	**Compound Heterozygous c.[916C > T] + [c.4433G > A]** **(p.[R306C] + [p.G1478E])**	+	+	–	+	+	–	–	–	–	–	+	3
**10**	**M**	**Compound Heterozygous c.[1960G > T] + [4559 T > C]** **(p.[E654X] + [L1520P])**	–	–	–	+	+	–	–	+	+	–	+	21
**11**	**F**	**Compound Heterozygous c.[3632 T > G] + not found 2nd mut** **(p.L1211R)**	–	+	–	+	+	–	+	–	–	+	–	12
**12**	**F**	**Recessive** **c.4559 T > C; (p.L1520P)**	–	–	+	+	+	–	–	+	–	–	–	21

(^a^) splicing mutation: unpredictable aminoacidic alteration; Flanagan et al. 2013 AmJHumGenet 10;192

**Table 2:** Patients with metabolic defects (MetD) and patients with undetected defects

	Sex	Genotype	Family History		Birth		HY Onset		Treatment		Follow-up			
			Hypoglycaemia	Diabetes (IFG/IGT)	At Term	LGA	< 72 h	Postneonatal	Dx responsiveness	Surgery	DM	Obesity	Neurological Impairment	Duration (years)
**1**	**F**	**GCK** **c.1363G > T; (p.V455S)**	–	–	+	+	NR		+	–	–	–	+	3
**2**	**M**	**HADH recessive c.706C > T; (p.R236Ter)**	–	–	+	–	–	–	+	–	–	–	–	4
**3**	**M**	**GLUD1** **c.978G > A; (p.R269H)**	+	–	+	–	–	+	+	–	–	+	–	17
**4**	**F**	**GLUD1** **c.820C > T; (p.R276C)**	+	–	+	–	–	+	+	–	–	–	–	16
**5**	**M**	**Undetected defect**	+	+	+	–	–	+	+	–	–	–	–	10
**6**	**F**	**Undetected defect**	–	+	+	–	+	–	+	–	–	+	–	18
**7**	**M**	**Undetected defect**	–	–	+	–	–	+	+	–	–	–	+	17
**8**	**M**	**Undetected defect**	+	+	+	–	+	–	+	–	–	–	–	4
**9**	**M**	**Undetected defect**	–	–	+	–	+	–	+	–	–	+	–	8
**10**	**M**	**Undetected defect**	–	–	+	–	–	+	+	–	–	+	–	20

*NR* not reported

## Results

Of all the 22 patients, 16 have received molecular diagnosis: 12 *ABCC8* mutations (54%), 2 *GLUD1* (9%), 1 *GCK* (4%) and 1 *HADH* (4%). Consequently, in this population the detection rate of genetic defects is 73%, higher than 45–55% reported by Rahman and others [5].

GMD were found in 7/12 families of ChD cases, 2/4 of MeD (both having *GLUD1* mutation) and 3/6 of unidentified defects; preterm birth was found in 6/12 ChD cases and in none of other categories; LGA was detected in 6/12 ChD cases and in only one MeD (with *GCK* mutation); time of presentation of HY in the first 72 h of life was found in 11/12 ChD and in 3/6 unidentified defects; Dx responsiveness was found in 6/12 ChD and in all MeD + unidentified defects subjects; diabetes in the follow-up was found in 4/12 ChD (2 subjects who had undergone pancreatectomy and 2 with late presentation); none in MeD and unidentified defects; obesity in the follow-up was found in 5/12 ChD, in 1/4 MeD and in 3/6 unidentified defects; neurological impairment in the follow-up was found in 4/12 ChD (subject number 5 epilepsy, subject number 7 epilepsy and severe developmental delay, subjects numbers 9 and 10 developmental delay), 1/4 MeD (this is G. V, described extensively below) and 1/4 unidentified defects (epilepsy).

We have performed ^18^F-DOPA PET-CT to 14/22 of described patients and we have always found diffused forms. A recent newborn with a focal form resolved by surgery (partial pancreatectomy) and four further cases are not included in this study due to the lack of three-year follow-up.

### Late-presentation cases

Family B. B.A. (male, 12-years old) has been referred to us because of asymptomatic fasting hyperglycemia occasionally detected. He was born preterm (35 weeks) and LGA (4150 g) from pregnancy characterized by GDM. B.A. and his brother, B. B, 17-years old, had presented neonatal HY and, during childhood, post-meal HY; also B.B. 3 years later, received a diagnosis of diabetes. Their mother, maternal grandmother and aunt presented diabetes treated with metformin. The mother’s first pregnancy was characterized by GDM and hesitated in a LGA newborn dead at birth. After excluding autoimmune diabetes, B.A. has been initially treated with a low glycemic index diet and later with sulfonylurea. Heterozygous mutation c.G4435 > A (p.Gly1479Arg) (already reported in literature as responsible of this phenotypic variability) [12] was detected in B.A., B.B. and their mother; the grandmother and the aunt refused genetic investigations. Data of B.A. and B.B. are reported in Table 1 (Patients number 1 and 2).

G.V. (female, 15 years old) has been referred to us due to an occasionally detected HY. She was born at term (40 weeks), LGA (4800 g) and was adopted at 2 years of age; neonatal neurological damage of unknown origin was reported. During childhood, she presented generalized epilepsy, treated with valproic acid, and moderate psychomotor impairment. Laboratory investigations, executed during HY (glucose value 42 mg/dL), showed hyperinsulinism (20,1 μUI/ml), while ammonium, lactic acid, cortisol, IGF-1, plasmatic aminoacids, acylcarnitines and urinary organic acids profiles were normal. V455L heterozygous mutation of Glucokinase gene (*GCK*) was detected; this mutation has not been yet described in literature. She started Dx-treatment at the dose of 8 mg/kg/die with a good response. Data of G.V. are reported in Table 2 (patient number 1).

### Flow-chart

**Fig. 1:**
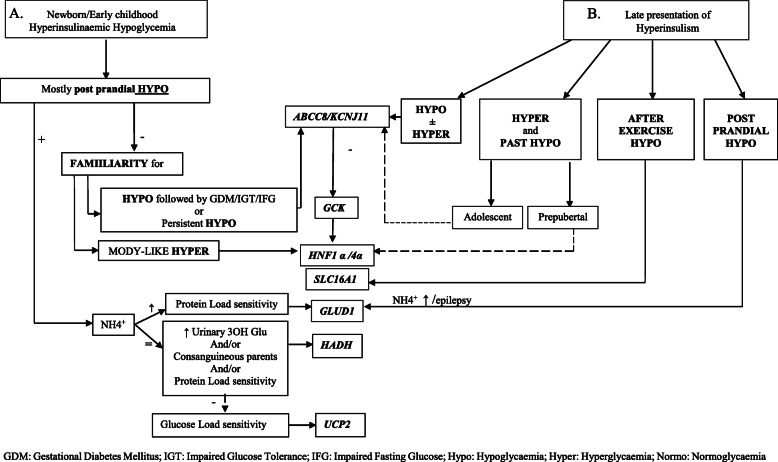
Clinical flow chart to diagnose CHI. GDM: Gestational Diabetes Mellitus; IGT: Impaired Glucose Tolerance; IFG: Impaired Fasting Glucose; Hypo: Hypoglycaemia; Hyper: Hyperglycaemia; Normo: Normoglycaemia

From the observation of the reported cases, particularly in consideration of late presentation cases, we tried to design a new CH diagnostic flow-chart (Fig. 1) expanding the diagnostic part of Maiorana’s algorithm by putting on the first step the age of presentation: section A refers to CH cases with precocious presentation while section B refers to CH cases with late presentation. In section A, the occurrence of post meal HY directs either to *GLUD1*, *HADH* or *UCP2* [16–19] defects, depending on the associated metabolic findings. The presence of a non-specific hypoglycemic pattern with positive HY family history can point toward *ABCC8/KCJN11* mutations; if they are not altered, firstly *GCK* and then *HNF1α/4α* should be tested, as already reported. In section B, the non-specific hypoglycemic pattern with hypo/hyperglycemia fluctuation points towards the *ABCC8/KCJN11* mutation: if not altered, firstly *GCK* and then *HNF1α/4α* should be tested. Mild hyperglycemia and the history of the previous HY point toward *ABCC8/KCNJ11* or *HNF1α/4α* mutations, especially in late onset cases. HY occurring after physical exercise directs toward *SCL16A1* mutation [20, 21]; post prandial HY, especially if associated with epilepsy or hyperammonemia, can suggest *GLUD1* mutation [16, 17].

## Discussion

Observations made on the 22 patients according to the literature are: a higher prevalence of familiar GMD, of LGA, of preterm birth and precocious HY during the first 72 h in ChD subjects; good Dx-responsiveness in MeD and unidentified defects, but only partial in ChD [22].

Literature concerning follow-up of CH cases generally focuses only on diabetes secondary to surgery: in our cases two subjects showed diabetes after subtotal pancreasectomy and the other two were the two brothers of the B. family who had the ABCC8 mutation. Interestingly 9/22 patients developed obesity during their follow up: 5 patients with ChD, 1 with GLUD1 and 3 with unidentified defects. This finding could be linked to ABCC8 role in susceptibility to obesity, as already reported in Type 2 Diabetes subjects [23], probably facilitated by hyperinsulinism; however, it is known that some *ABCC8* Single Nucleotide Polymorphisms seem to be protective against metabolic syndrome [24]. Furthermore, the presence of neurological impairment, reported in 4 subjects affected by precocious and severe forms of ChD, 1 with GCK (case G.V. above described) and 1 with unidentified defect, is in agreement with the literature.

CH diagnostic algorithms start from the metabolic investigations [16, 17, 25, 26] and proceed with molecular diagnosis and genotype-phenotype correlation. Dx trial was considered the crucial step in CH treatment: resistant patients must be treated with octreotide and need to be characterized by ^18^F-DOPA PET-CT, to address the appropriate surgery (near-total pancreatectomy for diffuse forms or confined excision for focal forms) [2, 6]. On the other hand, the most recent approach starts from the research of peculiar metabolic findings to guide the genetic screening, which is the fundamental step, rather than the Dx-responsiveness, to select patients for ^18^F-DOPA PET-CT [9]. As a matter of fact, these authors have found that, although rare, some focal forms could be Dx-responsive and could be completely solved by surgery. Consequently, ^18^F-DOPA PET-CT is indicated in case of one recessive paternal *ABCC8/KCNJ11* mutation in order to search for focal lesions, or when no mutation in the principal causative genes (ABCC8/KCNJ11, HNF1/4, GCK, HADH, SLC16A1, UCP2) is found, to search for atypical forms.

CH can occur throughout the entire childhood or adulthood. All available diagnostic algorithms don’t take into account late-presentation cases which could represent late forms but also misdiagnosed and mild forms. It is known that *ABCC8* mutations can cause CH, evolving into hyperglycemia and gestational diabetes [10, 13, 27, 28]. Concerning our cases, the family of B.A. and B.B. is similar to a case previously described with a c.4435G > A mutation [12].

GCK activating mutations cause a heterogeneous phenotype: the severity of the symptoms and the age of presentation present substantial variations between affected individuals, even within the same family [22, 23]. For this reason, testing *GCK* gene is recommended in subjects with a non-specific pattern of HY. In our case, *GCK* c.1363G > T mutation has not been reported yet.

Our experience suggests that the algorithm to better diagnose CH should consider a wider approach, starting from the age of the first detection. When CH develops into diabetes, HY usually occurred during the early childhood and, in subsequent years, occurred in post-prandial time, after high glycemic index meals. In presence of family history of HY and/or Impaired Glucose Tolerance, Impaired Fasting Glucose or GDM, the hypothesis of *ABCC8/KCNJ11*, *HNF1A* or *HNF4A* gene mutations can be formulated. In particular, in *ABCC8* gene mutations, hyperglycemia generally starts in adolescence, while in *HNF1α/4α* in prepubertal age [10–14].

As affirmed by others, ^18^F-DOPA PET-CT should be performed when a focal form is suspected in the presence of Dx-unresponsiveness (although some rare focal forms respond to diazoxide) and if no mutation is detected at all; in these patients images provided by ^18^F-DOPA PET-CT can address the choice of surgical treatment [8, 9, 27, 29]. Thus, in late presentation cases, we can assume that ^18^F-DOPA PET-CT should be performed only to exclude insulinoma, since it can occur at any age, but is very rare in pediatric age, and could represent a manifestation of Multiple endocrine neoplasia type 1 (MEN1) [30–32]. Insulinoma could either be detected by ^18^F-DOPA PET-CT or (68) Ga-DOTATATE [32]. Without a suspect of Insulinoma, it is difficult that ^18^F-DOPA PET-CT should be indicated for subjects with late presentation. *ABCC8/KCNJ11* late diagnosed cases reported in literature are mostly due to dominant mutations which determine a diffused form; likewise MeD forms are all diffused [10–14].

In our cases, the rate of positivity of genetic tests is 73% (16/22), significantly higher than reported in literature (45–50%) [5]. It probably depends on a strict collaboration between experts of diabetes, metabolism and neonatal pathology in our University Hospital.

## Conclusion

In conclusion, since CH may start not only in the neonatal period or infancy, but also in childhood or even later, it may be useful to present a diagnostic flow-chart that includes also these cases by considering the glycemic alterations of the whole family, both in the present and in the past.

## Data Availability

All data generated during this study are included in this published article and its supplementary information files.

## Abbreviations

CH: Congenital Hyperinsulinism
HY: Hypoglycemia
ChD: Channel defect
MeD: Metabolic defect
SUR1: Sulfonylurea Receptor 1
Dx: Diazoxide
GDM: Gestational Diabetes Mellitus
GMD: Glucose metabolism derangements
LGA: Large for gestational age
